# Effect of GLP-1 receptor agonists at doses for obesity management on muscle health: systematic review and meta-analysis of randomized controlled trials (RCTs)

**DOI:** 10.1038/s41366-026-02118-y

**Published:** 2026-06-19

**Authors:** Ligia Patricia Laverde, Oscar Mauricio Muñoz-Velandia, Diana Alfonso, Ana María Gómez Medina

**Affiliations:** 1 Division of Endocrinology, Hospital Universitario Fundacion Santa Fe, Bogotá, Colombia; 2 Department of Internal Medicine, Pontificia Universidad Javeriana, Bogotá, Colombia; 3 Department of Internal Medicine, Hospital Universitario San Ignacio, Bogotá, Colombia

**Keywords:** Obesity, Medical research

## Abstract

**Introduction:**

The impact of GLP-1 receptor agonists (GLP1-RA) treatment on lean mass and body composition in patients with obesity is not fully understood.

**Methodology:**

Systematic review and meta-analysis of RCTs including patients with obesity treated with GLP1-RA at obesity doses, compared with placebo. Search was conducted in PubMed, Embase, and LILACS in March 2025. A meta-analysis was performed using a random-effects model to evaluate the change in lean mass as a proportion of total weight, the absolute and relative changes in lean mass, and adverse effects.

**Results:**

Seven studies (821 patients) were included in the analysis. GLP1-RA significantly improved lean mass as a proportion of total weight, with an observed increase of 1.81% (95% CI: 1.1– 2.52; *p* < 0.00001; *I*² = 7%). However, a significant decrease was observed in the absolute change in lean mass (−1.74 kg; 95% CI: −3.04 to −0.45; *p* < 0.00001; *I*² = 98%) and in the percentage of lean mass (−3.06%; 95% CI: −5.10 to −1.02; *p* < 0.00001; *I*² = 98%). Semaglutide demonstrated the most significant reduction in absolute lean mass, with a loss of −5.44 kg (95% CI: −7.07 to −3.81; *p* < 0.00001).

**Conclusions:**

There is an improvement in body composition due to an increase in lean mass as a proportion of total weight, an effect that could be amplified with the use of semaglutide. Our results suggest that lean mass loss should not be considered a limitation for the use of these drugs in patients with obesity. However, it is essential to accompany drug treatment with nutritional and physical exercise interventions to preserve or improve muscle mass and optimize clinical outcomes.

## Introduction

Obesity is a chronic disease whose prevalence continues to increase worldwide. An estimated 16% of adults were affected by 2022 [1]. Obesity represents a significant burden of morbidity and mortality, causing more than 5 million deaths each year [2]. The most common comorbidities, which contribute to people with obesity being a public health priority, are chronic noncommunicable diseases such as cardiovascular disease, various types of cancer, Alzheimer’s disease, and type 2 diabetes mellitus [3].

Glucagon-like peptide-1 receptor agonists (GLP1-RA) have emerged as an effective pharmacological treatment option for people with obesity [3]. These drugs mimic the action of endogenous incretins, promoting satiety and reducing appetite. This leads to a sustained decrease in caloric intake and clinically significant weight loss ranging from 5% to 21%, depending on the drug and duration of use [4, 5]. As their use expands, evaluating not only the amount of weight lost but also the change in body composition—particularly in relation to muscle mass—becomes important, given its significance for metabolism, physical function, and quality of life [6].

Weight loss, particularly when rapid or pharmacologically induced, may be accompanied by a reduction in lean body mass, which increases the risk of sarcopenia [6]. The coexistence of obesity and sarcopenia, known as sarcopenic obesity, is associated with impaired metabolic control, increased risk of falls, fractures, cardiovascular events, hospitalization, and mortality [7]. The potential impact of GLP1-RA therapies on muscle mass loss has raised concerns. Lean mass differs from muscle mass in that lean (fat-free) mass includes not only skeletal muscle, but also organs, bones, body fluids, and hydration status [8].

According to recent meta-analyses of studies on patients with overweight, obesity, and/or diabetes, GLP1-RA have been shown to be effective in reducing total body weight and fat mass [9]. However, these analyses have also indicated that these medications are associated with a significant reduction in lean mass. It is estimated that approximately 25% of the weight lost corresponds to lean mass [9]. However, the relative change in lean mass (percentage from baseline) was not statistically significant.

Other recently published meta-analyses revealed a modest reduction in lean mass compared to the control group (mean difference = –1.02 kg; 95% CI: –1.46 to –0.57 kg) [10]. Additionally, changes in the percentage of lean body mass were comparable between users and non-users of GLP1-RA (mean difference = –0.10%; 95% CI:–1.31% to 1.11%) [10].

However, these analyses included studies that used GLP1-RA at doses approved for diabetes treatment. This may have underestimated the true effect on lean mass reduction when GLP-1 RA are used at the doses indicated for obesity treatment, since the doses indicated for diabetes treatment are lower than those indicated for obesity treatment [11].

Given the conflicting evidence, we conducted a systematic review and meta-analysis that included RCTs in participants with obesity (with or without diabetes) treated with GLP1-RA at doses exclusively indicated for obesity, compared with placebo, to assess their effects on relative lean body mass and absolute lean body mass.

## Methods

This review has been prepared in accordance with the PRISMA (Preferred Reporting Elements for Systematic Reviews and Meta-Analyses) guidelines [12]. The protocol was approved by the ethics committee of Hospital San Ignacio and the Pontificia Universidad Javeriana (Approval code 007-2025). This systematic review and meta-analysis were conducted according to a prespecified protocol registered in PROSPERO (CRD420251070012).

### Search strategy and selection criteria

We included randomized clinical trials (RCTs) that evaluated adults diagnosed with obesity (defined as a body mass index greater than 30) with or without type 2 diabetes mellitus if they compared treatments based on GLP1-RA at approved obesity doses vs. placebo. The trials included the following specific therapies and doses: liraglutide (3 mg daily), semaglutide (2.4 mg weekly), and tirzepatide (5, 10, or 15 mg weekly). Trials evaluating survodutide and retatrutide were excluded, as these agents have not yet been approved by the FDA for the treatment of obesity.

To be considered eligible, studies had to report at least one of the outcomes of interest related to body composition, lean mass, and muscle function, including: percentage change in absolute lean mass, percentage change in lean mass as a proportion of total weight, and change in hand strength. These outcomes had to be assessed using either X-ray densitometry (DXA), computed tomography, magnetic resonance imaging, or electrical bioimpedance.

Studies evaluating patients with type 1 diabetes, pregnant women, patients undergoing bariatric surgery, and those using methods other than those mentioned for measuring body composition were excluded from the analysis. A search was conducted in the PubMed (MedLine), EMBASE (Elsevier), and LILACS databases using the following terms: “Obesity,” “glucagon-like peptide 1 receptor agonists,” “liraglutide,” “semaglutide,” “tirzepatide,” “anti-obesity agents,” “body composition,” “anthropometry,” “body weight,” “electric impedance,” “muscle, striated,” “muscle, skeletal,” “magnetic resonance imaging,” “absorptiometry, photon,” and “hand strength.” No language restrictions were applied. The search was conducted between January 1, 2004, and April 1, 2025, with the start date chosen based on the year of publication of the first studies on liraglutide in people with diabetes. The specific search strategies for each database are detailed in Appendix 1.

**Fig. 1:**
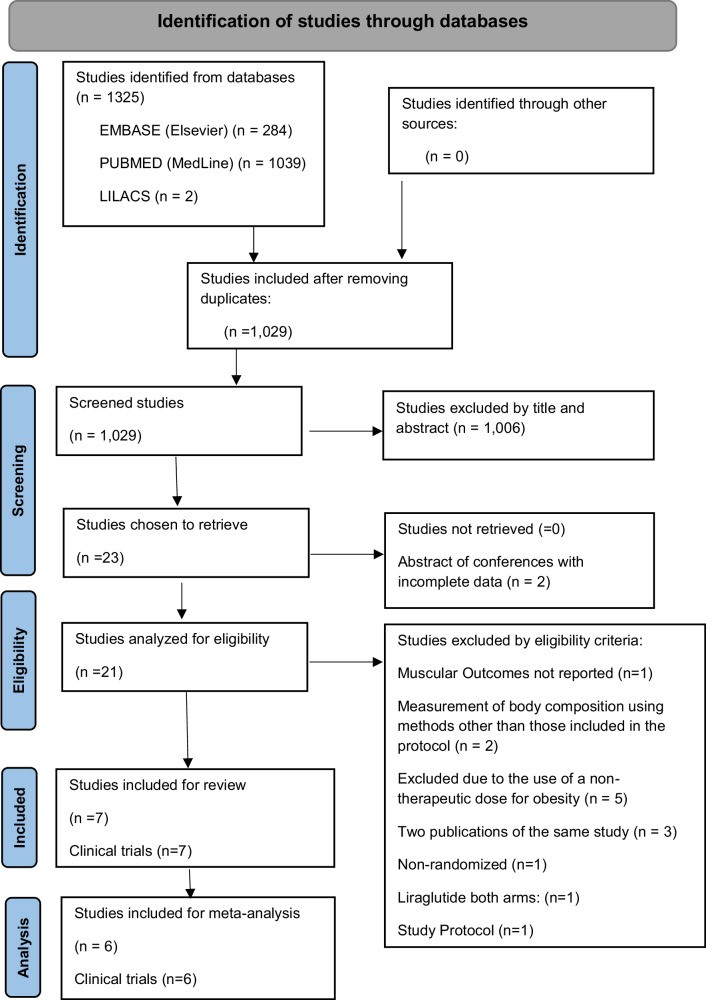
PRISMA flowchart. Diagram illustrating the process of study identification, screening, eligibility assessment, and inclusion in the systematic review and meta-analysis.

For the selection process, two authors (LPL, DA) independently reviewed the titles and abstracts of the retrieved records and evaluated the full texts of potentially eligible articles. Discrepancies were resolved by consensus or by involving a third reviewer (OMM). Figure 1 presents the PRISMA flow diagram, which illustrates the selection process.

#### Information extraction and risk of bias assessment

Outcome data were extracted independently by two authors (LPL, DA) using a standardized method. Discrepancies were resolved through consensus.

To perform the meta-analysis with studies that did not report the change in lean mass as a proportion of total weight, this was calculated from lean mass in kilograms, or as a percentage of total weight at baseline and at the end of the interventions. In studies that did not report the standard deviation (SD) of the change in lean mass or other outcomes, this was estimated from the available data (standard errors, confidence intervals, or interquartile ranges), following the methods described by Wan et al. [13] and Luo et al. [14]. These transformations enabled the incorporation of studies with incomplete data in the meta-analysis, aligning with the guidelines outlined in the Cochrane Handbook for Systematic Reviews.

The risk of bias in RCTs was assessed independently by two authors (LPL, DA) using the Cochrane Collaboration’s RoB tool [15]. Discrepancies were resolved by consensus or by a third assessor (OMM).

#### Synthesis of the assessment and evaluation of the certainty of the evidence

The outcomes related to absolute lean mass and lean mass proportion were synthesized using the standardized mean difference (SMD) using in the meta-analysis a random-effects model. Additionally, a subgroup analysis was planned a priori according to the medication used and the follow-up time (less vs. greater than 52 weeks).

Summary measures are presented in forest plots or as part of the narrative synthesis. Heterogeneity was assessed using the *I*² statistic, which allows for the estimation of the proportion of variation in results attributable to differences between studies and not to chance. The analysis was conducted using the Cochrane Collaboration’s RevMan version 5.4® software.

A publication bias analysis using a funnel plot was not performed because fewer than ten randomized studies were included in the meta-analysis, which could limit the reliability and interpretability of the analysis.

The certainty of the evidence for each outcome was assessed using the GRADE methodology [16]. The Summary of Findings (SoF) was generated using the GRADEpro tool (https://gdt.gradepro.org/app/).

## Results

The selection process for studies to include in the systematic review is outlined in Fig. 1. Finally, seven RCTs [17–23] were included in the review, involving a total of 821 patients. Table 1 presents the clinical characteristics of the patients included in each study. Five RCTs evaluated liraglutide, four of them for less than 52 weeks and one for more than 52 weeks. One evaluated semaglutide [18], and another evaluated tirzepatide [19].

**Table 1:** Characteristics of included studies.

Author, year	Intervention (Agent, dose)	Control	No. of subjects (I, C)	Age (Years) (mean, SD)	Female	Baseline BMI (kg/m2) (mean, SD)	Glucose tolerance status	Comorbidities	HbA1c ((mean, SD)	Treatment duration (weeks)	Weight loss	Method to evaluate body composition	Change in total fat mass	% Change in VAT
Astrup 2012 [17]	LIRA 3 mg per day	PL	LIRA: 15, PL: 14	LIRA: 45.9 (10.7) PL: 45.9 (10.3)	LIRA: 75% PL: 75%	LIRA: 34.8 (2.8) PL: 34.9 (2.8)	DM LIRA: 4.3% Placebo: 4.1% PreDM LIRA: 29% PL: 32.7%	HT: LIRA: 12% PL: 28% DL: LIRA: 8% PL: 4%	NR	20	LIRA - 7.2 kg, PL −2.8 kg.	DXA, TC	LIRA −15.4% (2.6), PL −11.9%	LIRA, −20.3%(6.0), PL13.8%(5.7)
Kadouh 2020 [21]	LIRA 3 mg day	PL	LIRA: 19, PL :21	LIRA 42 [32, 51], PL: 37 (26, 51)	LIRA: 94%, PL: 85	LIRA: 37.2 (33.6, 41.0), Placebo: 34.6 (33.4, 38.9)	NR	NR	NR	16	LIRA: −5.3 kg (5.2-6.8), PL: −2.5 kg [0.1–4.2]	DXA	LIRA: –2 (–3.15, –1.45), Placebo: –0.4% (–1.75, 0.275)	NR
Lundgren 2021 [23]	LIRA 3 mg day + usual activity LIRA (3.0 mg) daily+ exercise	PL+ usual activity	LIRA: 49, LIRA+ Exercise: 49 PL:49	43(12)	64	32.6(2.9)^a^	DM 0% PreDM: LIRA 3% PL 5%	NR	5.3	52	LIRA: −0.7 kg LIRA+ Exercise: −3.4 kg PL: +6.kg	DXA	LIRA: −1.6 (−4.2, −1.1) kg LIRA+ Exercise: −4.3 kg (−6.8, −1.8) placebo: + 3 kg	NR
Neeland, 2021 [22])	LIRA 3.0 mg day	PL	LIRA: 73 PL: 55	LIRA: 49.6(9.8) Placebo: 50.9(8.8)	LIRA: 92%, PL: 93%	LIRA 37.2(6.0) PL 38.1(6.1)	DM 0% PreDM LIRA 3%, PL 5%	HT: LIRA 41%, PL 36%, DL: LIRA 20%, PL 29%	NR	40	LIRA: −6.75 kg (5.35) PL −1.3 kg (4.79)	MRI	LIRA −3.76 L (2.87) PL −0.42 L (2.92)	LIRA: −0.53 L (0.43) PL -0.1 L (0.52)
Wilding 2021 [18]	SEMA 2.4 mg weekly	PL	SEMA 95, PL 45	SEMA: 50(12), PL 52(13)	SEMA: 75.8%, PL 75.6%	SEMA: 34.8(3.6), PL: 35(3.6)	DM 0% preDM SEMA 45.4% PL 40.2%	DL SEMA 38.2%, PL: 34.5% HT: SEMA 36.1% PL 35.7% OSA: SEMA 12% PL 10.8%	SEMA: 5.7%(0.4), PL 5.7%(0.3)	68	SEM −15.3 kg, PL. −2.4 kg	DXA	SEMA −8.36 kg PL −1.37 kg	SEMA −0.36 KG, PL: -0.1 KG
Elkind-Hirsch 2022 [20]	LIRA 3 mg day	PL	LIRA (55) PL (27)	LIRA: 31(0.8), PL: 32(1.2)	LIRA and PL: 100% female	LIRA: 41.6(1.1), PL: 43.9(1.7)	DM 0%	PCOS 100%	LIRA: 5.5+/−0.05, PL: 5.5+/−0.08	32	LIRA: - 6.3 kg, PL – 1.4 KG	DXA	LIRA −4.68 kg^b^, PL: −0.89 Kg	NR
Jastreboff 2022 [19]	TIRZE 5, 10, 15 mg weekly 1:1	PL	255^c^	TIRZE 5 mg: 45.6, TIRZE 10 mg: 44.7, TIRZE 15 mg: 44.9, PL: 44.4	TIRZE: 67.5% PL: 67.8%	TIRZE 5 mg: 37.4(6.6), TIRZE 10 mg: 38.2(7.0), TIRZE 15 mg: 38.1(6.6), PL: 38.2(6.8)	DM 0% PreDM TIRZE 5 mg: 39.2%, TIRZE 10 mg: 41.2%, TIRZE 15 mg 40.2%, PL: 42%	NR	TIRZE 5 mg: 5.6 ± 0.36%, TIRZE 10 mg: 5.6 ± 0.37%, TIRZE 15 mg: 5.6 ± 0.41%, PL: 5.6 ± 0.38%	72	TIRZE 5 mg: −16.1 kg TIRZE 10 mg: −22.2 kg TIRZE 15 mg: −23.6 kg PL: −2.4 kg	DXA	TIRZE −33.9% PL −8.2%	NR

*LIRA* liraglutide, *PL* placebo, *DM* diabetes mellitus, *PreDM* prediabetes, *HT* hypertension, *DL* dyslipidemia, *NR* not reported, *DXA* dual-energy X-ray absorptiometry, *CT* computed tomography, *SEMA* semaglutide, *OSA* obstructive sleep apnea, *TIRZE* tirzepatide, *PCOS* polycystic ovary syndrome, *MRI* magnetic resonance imaging, *VAT* visceral fat, *L* liters.

^a^The Lundgreend study does not break down age, sex, glycated hemoglobin, or BMI for each intervention group.

^b^Calculated based on data provided in the Elkind study.

^c^The Jastreboff study does not specify how many of these 255 participants received each individual dose of tirzepatide (5 mg, 10 mg, or 15 mg) or how many were in the placebo group within this specific DXA subgroup.

All studies included counseling for both groups on lifestyle interventions (diet and physical activity) unless otherwise stated in Table 1.

The average age of participants in the studies ranged from 31 to 51 years. It should be noted that the majority of studies excluded diabetic patients, while the semaglutide [18] and tirzepatide [19] studies included more than 30% of prediabetic patients. The average baseline glycated hemoglobin varied between 5.3% and 5.7%. The most common comorbidities were dyslipidemia, hypertension, and polycystic ovary syndrome.

### Assessment of risk of bias

Following a thorough evaluation using the ROB tool [15], all studies were deemed to be at low risk of bias. Table 2 presents the risk of bias assessment for each study. Two studies had a risk of bias associated with selective reporting of outcomes [18, 19].

**Table 2:**
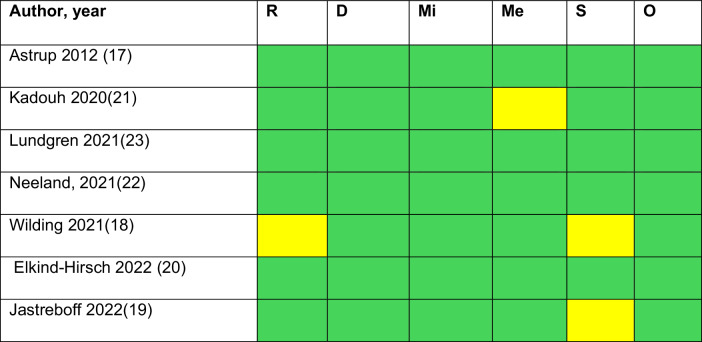
Assessment of risk of bias (RoB): randomized studies.

R: Bias arising from the randomization process; D: Bias due to deviations from intended interventions

Mi: Bias due to missing outcome data; Me: Bias in measurement of the outcome; S: Bias in selection of the reported result

O: Overall risk of bias

Result:

Low  Some concern  High

### Change in lean mass as a proportion of total weight (%)

**Fig. 2:**
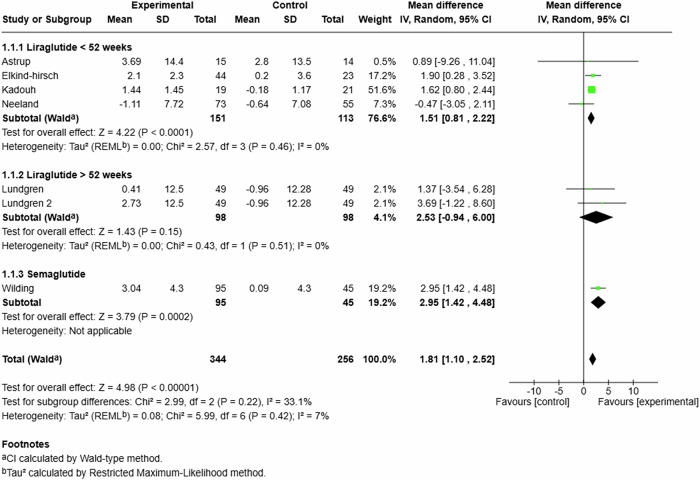
Change in lean body mass relative to total weight. Forest plot of changes in lean body mass relative to total body weight following GLP-1 receptor agonist treatment.

The change in lean body mass as a proportion of weight was evident in the overall analysis and within subgroups according to medication and follow-up time (liraglutide <52 weeks, liraglutide >52 weeks, and semaglutide). In the studies evaluating liraglutide for <52 weeks an increase of 1.51% (95% CI: 0.81–2.22; *P* < 0.0001; *I*² = 0%) was evident. After 52 weeks of treatment with liraglutide, the change in lean mass as a proportion of total weight was higher 2.53% (95% CI: −0.94–6; *P* = 0.15). Semaglutide demonstrated a 2.95% improvement (95% CI: 1.42–4.48; *P* = 0.0002). The combined result was a 1.81% increase (95% CI: 1.1–2.52; *P* < 0.00001; *I*² = 7%), with no significant heterogeneity (Fig. 2). The certainty of the evidence for this outcome was high (Supplementary Table 1).

### Percentage change in lean mass (%)

**Fig. 3:**
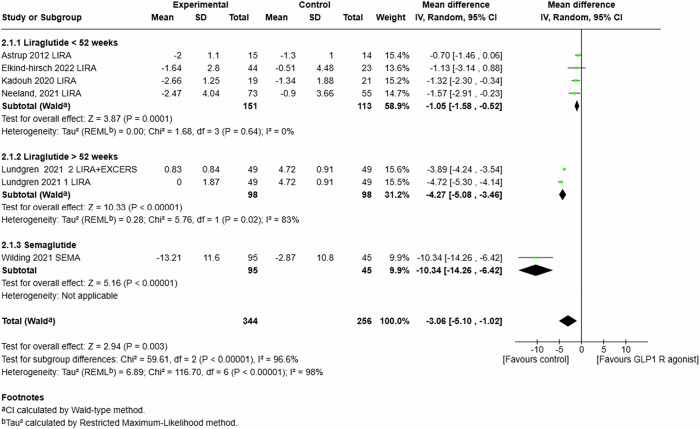
Changes in lean body mass %. Forest plot showing the effect of GLP-1 receptor agonists on percentage changes in lean body mass compared with control groups.

All analyses indicated a decrease in lean body mass, with semaglutide demonstrating a more significant effect. In the studies with liraglutide for <52 weeks, there was a −1.05% reduction (95% CI: −1.58 to −0.52; *P* = 0.0001; *I*² = 0%). After following the same medication for a period of more than 52 weeks, the results showed a decrease of −4.27% (95% confidence interval: −5.08 to −3.46; *P* < 0.00001; *I*² = 0%). Semaglutide demonstrated the most significant reduction, with a percentage decrease of −9.9% (95% CI: −14.26 to −6.42; *P* < 0.00001). The combined effect was −3.06% (95% CI: −5.10 to −1.02; *P* < 0.00001; *I*² = 98%), with the type of medication used being the main source of heterogeneity (Fig. 3). The certainty of the evidence for this outcome was high (Supplementary Table 1).

### Absolute change in lean mass (KG)

**Fig. 4:**
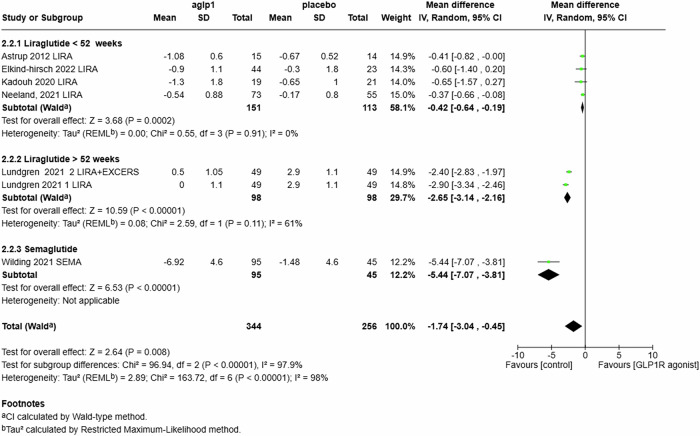
Changes in lean body mass (KG) (absolute change). Forest plot showing the effect of GLP-1 receptor agonists on absolute changes in lean body mass (kg) compared with control groups.

Consistent with the findings regarding the percentage change in lean mass, the comprehensive analysis and the analysis within each of the subgroups indicated an absolute reduction in kilograms of lean mass, with semaglutide demonstrating a more pronounced effect. In the studies with <52-weeks with liraglutide, the difference was −0.41 kg (95% CI: −0.82 to −0.0; *P* = 0.0002; *I*² = 0%). With the same medication and follow-up for over 52 weeks, there was a reduction of −2.65 kg (95% CI: −3.14 to −2.16; *P* < 0.0001; *I*² = 61%). Semaglutide demonstrated the most significant absolute loss, with a mean difference of −5.44 kg (95% CI: −7.07 to −3.81; *p* < 0.00001). The combined effect was −1.74 kg (95% CI: −3.04 to −0.45; *p* < 0.00001; *I*² = 98%), with follow-up time and medication used being important sources of heterogeneity (Fig. 4). The certainty of the evidence for this outcome was high (Supplementary Table 1).

Unfortunately, data from the SURMOUNT-1 study on tirzepatide [19] could not be included in the meta-analysis because results were not reported in the subgroup of patients analyzed with DXA. However, it is reported that the change in lean body mass percentage in the tirzepatide group was −10.9% vs. −2.0% in the placebo group with an estimated treatment difference of −8.3% (CI: −10.6, −6.1). Participants treated with tirzepatide exhibited a percentage reduction in fat mass approximately three times greater than the reduction in lean mass, resulting in an overall improvement in body composition.

### Adverse events

The most common adverse events were gastrointestinal, including nausea, vomiting, diarrhea, and constipation. These events were generally mild and transient. The incidence was higher with semaglutide (74.2% vs. 47.9% with placebo) [18], liraglutide (53% vs. 8%), and tirzepatide (up to 81.8% vs. 72%) [19]. Discontinuations due to adverse events occurred in 7.0% of patients treated with semaglutide, 9% of patients treated with liraglutide, and up to 7.1% of patients treated with tirzepatide. Serious events were rare, with a frequency of less than 10%, including cholelithiasis (up to 2.6%) and mild pancreatitis [17, 18, 23]. The GRADE evaluation indicates a moderate certainty of the evidence for this outcome, considering potential bias from selective reporting of outcomes.

## Discussion

In this study, we evaluated changes in lean mass in patients with obesity treated with GLP1-RA at doses approved for obesity management. Our findings indicate that while there is a significant reduction in absolute lean mass, the percentage of lean mass relative to total weight increases, with this effect being more pronounced with semaglutide.

A modest decrease in absolute lean mass has been reported in two previous meta-analyses [9, 10]. These meta-analyses included studies involving patients with diabetes and overweight, who were treated with low doses of GLP-1 RA. In our analysis, by focusing exclusively on the higher doses indicated for obesity, we observed a more pronounced absolute change, as the potency of GLP1-RA agonists is dose-dependent [11].

Unlike previous meta-analyses that found no statistically significant change in the percentage of lean mass, our meta-analysis shows an improvement in body composition due to an increase in lean mass relative to total weight. This discrepancy can be attributed to the enhanced fat mass loss, which results in a favorable shift in body composition. Therefore, even if lean mass is lost in absolute terms, the net balance favors a healthier body composition, which may represent a clinical benefit [6].

To our knowledge, this is the first meta-analysis to specifically evaluate the effect of GLP1-RA on body composition and lean mass at doses for obesity, avoiding the possible attenuation of the effect observed with lower doses for diabetes.

In the subgroup analysis of studies that evaluated change in lean mass as a proportion of total weight (%), our findings suggest that, despite the absolute reduction in lean mass, the relative proportion of lean mass to total body weight increases significantly with the use of GLP1-RA, particularly with semaglutide. This finding indicates that fat mass loss exceeds lean mass loss, suggesting an improvement in body composition. The lack of heterogeneity in the liraglutide analyses at <52 weeks and in the overall analysis (*I*² = 7%) reinforces the consistency of this effect. While the results of the study on liraglutide use over 52 weeks showed a non-significant change, it should be noted that this finding is based on a single study with a reduced number of patients, which limits the interpretation of the results.

In contrast, the analysis of the percentage change in lean mass (%) revealed a reduction in the percentage of lean mass from baseline in all subgroups. The reduction was more pronounced with semaglutide (−9.9%). These results indicate that, while the proportion of lean mass relative to body weight may improve, absolute lean mass is reduced. This finding underscores the need for complementary strategies, such as resistance exercise and adequate protein intake during GLP1-RA therapy [23]. The significant heterogeneity of the combined analysis (*I*² = 98%) may be attributable to differences in the study populations, intervention duration, and body composition measurement methods. However, the direction of the effect is consistent in magnitude and direction across all studies.

As indicated by the aforementioned data, the analysis of absolute change in lean mass (kg) revealed an absolute reduction in all subgroups. Semaglutide demonstrated the most significant weight loss, with a mean reduction of −5.44 kg over 52 weeks. Liraglutide, administered for more than 52 weeks, showed a mean loss of −2.65 kg, while the <52-week regimen resulted in a mean loss of −0.41 kg. The greater reduction observed with semaglutide could be attributed to its increased potency and total weight loss, as previously described in studies [24]. Tirzepatide has a more pronounced effect on lean mass loss (−10.9%) [19], but it could not be included in the meta-analysis because no disaggregated data for each subgroup were reported in SURMOUNT-1. Although there is some heterogeneity in the analyses of liraglutide over 52 weeks (*I*² = 61%) and overall (*I*² = 98%), the direction of the effect is consistent. These findings highlight the potential for clinically significant muscle loss during treatment with GLP1-RA, emphasizing the importance of monitoring lean mass as a primary outcome in the treatment of patients with obesity.

When lean mass loss is analyzed as a proportion of total body weight loss, our findings are consistent with those of a recent meta-analysis [25], which reported that approximately 30% of total weight loss with GLP-1 RA therapy corresponds to lean mass—a proportion comparable to that observed after bariatric surgery. However, among the studies included in our meta-analysis, this proportion varied according to the specific GLP-1 RA used. For instance, with liraglutide, lean mass accounted for 14–22% of total weight loss; with semaglutide, this proportion reached up to 45%; and with tirzepatide, approximately 26% of total body weight loss was attributable to lean mass.

Lean mass is widely used as a surrogate for muscle mass and has therefore been evaluated in most clinical studies. Although imaging-based metrics, including MRI-derived muscle volume and *z*-scores, may offer a more refined assessment of muscle outcomes, evidence using these approaches remains limited.

The SURPASS-3 MRI subanalysis assessed muscle outcomes using muscle volume and MRI-derived *z*-scores [26]. This study was not included in our meta-analysis because our predefined muscle outcomes were lean mass and muscle function. Nonetheless, its findings provide relevant complementary evidence. In SURPASS-3 among patients with type 2 diabetes mellitus treated with tirzepatide (5, 10, and 15 mg) compared with insulin degludec, significant reductions in muscle volume (−0.64 L; 95% CI −0.74 to −0.54) and muscle *z*-score (−0.22; 95% CI −0.29 to −0.15) were observed, findings that are consistent with those reported in SURMOUNT-1.

In addition, the included studies did not evaluate functional outcomes, such as muscle strength or physical capacity. As a result, the clinical interpretation of the observed changes is limited. Further studies are necessary to assess functional muscle outcomes in these patients, as functional decline is known to occur earlier and progress more rapidly than changes in lean or muscle mass [27]. Therefore, evaluating functional outcomes would provide valuable insight into the clinical significance of these findings.

The results of this meta-analysis should be interpreted with the understanding that the studies included run-in periods in which candidates adhered to calorie-restricted diets and physical activity before starting GLP1-RA medications and then continued lifestyle intervention measures. This is important for their application in clinical practice.

The study revealed that patients assigned to the placebo group also experienced a loss of lean mass, despite receiving lifestyle interventions, including dietary and exercise counseling; this reduction is likely attributable to the physiological age-related decline, which has been reported to range between approximately 0.3% and 1% per year [27]. This decline could have been more pronounced in the absence of counseling on a healthy diet and exercise. In several studies, these patients gained weight and fat mass, leading to a deterioration in their body composition. This suggests that the effect of GLP1-RA may be more significant in these patients.

Several new molecules are currently under investigation and have shown promising results. Among them are retatrutide, a “triple agonist” targeting GIP (glucose-dependent insulinotropic peptide), GLP-1 (glucagon-like peptide-1), and glucagon receptors, and survodutide, a dual GLP-1 and glucagon agonist [8, 28]. Although these agents demonstrate substantial weight-loss effects, they were not included in our meta-analysis because they do not yet have FDA approval for the treatment of obesity. Moreover, current studies have not evaluated their impact on muscle-related outcomes—an aspect that will be essential to assess as these drugs move closer to clinical use.

There are some limitations in our study that need to be recognized. Despite attempts to contact the study authors, we were unable to obtain complete data for tirzepatide. As a result, the extrapolation of results to this drug is limited. Similarly, the number of patients and studies with semaglutide is limited, so further clinical trials with these drugs are needed to confirm our findings.

An additional limitation of the study is that information about functional muscle outcomes were not found. Controlled studies are needed. Another limitation is that heterogeneity of the estimate persists in some outcomes despite subgroup analyses, which were planned by separating the drugs as they have different effect intensities and separating the intervention time as this can generate different results. Other potential sources of heterogeneity may include differences in measurement methods and the ages of the participants.

While the change in lean mass proportion was statistically significant, it may not be clinically significant. However, it could be interpreted as a “no net deterioration” in the context of body composition and lean mass/total weight ratio. Due to the limited number of studies included, it was not possible to perform a formal analysis of publication bias. Finally, the results of this study pertain exclusively to non-diabetic patients, and most of the included studies were conducted in relatively young populations with a low burden of comorbidities. Therefore, the effects in patients with diabetes, as well as in older and multimorbid populations, should be evaluated in future studies, as these individuals are at high risk for muscle deterioration, underscoring the clinical importance of investigating this outcome in these populations.

## Conclusions

Our study indicates that there is an improvement in body composition due to an increase in lean mass as a proportion of total weight, an effect that could be amplified with the use of semaglutide. GLP1-RA used at doses for obesity has been associated with an absolute reduction in lean mass; however, this is less than the loss of fat mass, resulting in an improvement in body composition. We suggest that lean mass loss should not be considered a limitation for the use of these drugs in patients with obesity. However, it is essential to accompany drug treatment with nutritional and physical exercise interventions to preserve or improve muscle mass and optimize clinical outcomes.

## Supplementary information

Supplementary table

## Appendix 1

**PUBMED**

(Obesity[MeSH Terms]) AND ((Glucagon-Like Peptide-1 Receptor Agonists[MeSH Terms]) OR (Gastric Inhibitory Polypeptide/agonists[MeSH Terms]) OR (Liraglutide[MeSH Terms]) OR (Semaglutide[Supplementary Concept]) OR (Dulaglutide[Supplementary Concept]) OR (Tirzepatide[MeSH Terms]) OR (Exenatide[MeSH Terms]) OR (Lixisenatide[Supplementary Concept]) OR (Anti-Obesity Agents[MeSH Terms])) AND ((Body Composition[MeSH Terms]) OR (Anthropometry[MeSH Terms]) OR (Body Weight[MeSH Terms]) OR (“Electric Impedance”[MeSH Terms]) OR (“Muscle, Striated”[MeSH Terms]) OR (“Muscle, Skeletal”[MeSH Terms]) OR (“Magnetic Resonance Imaging”[MeSH Terms]) OR (“Absorptiometry, Photon”[Mesh]))AND (Hand Strength [MeSH Terms]) Filters: Full text, Clinical Study, Clinical Trial, Clinical Trial, Phase I, Clinical Trial, Phase II, Clinical Trial, Phase III, Clinical Trial, Phase IV, Comparative Study, Controlled Clinical Trial, Randomized Controlled Trial, from 2004 – 2025

**EMBASE**

‘obesity’/exp AND (‘glucagon like peptide 1 receptor agonist’/exp OR ‘liraglutide’/exp OR ‘semaglutide’/exp OR ‘dulaglutide’/exp OR ‘tirzepatide’/exp OR ‘exendin 4’/exp OR ‘lixisenatide’/exp) AND (‘bioelectrical impedance analysis’/exp OR ‘body composition’/exp OR ‘dual energy X ray absorptiometry’/exp OR ‘lean body weight’/exp OR ‘striated muscle’/exp) AND ‘clinical trial’/exp

**LILACS**

(obesity OR obesity) AND (“GLP-1 receptor analogs” OR “glucagon-like peptide-1 receptor agonists” OR liraglutide OR semaglutide OR dulaglutide OR tirzepatide OR exenatide OR lixisenatide OR “gastric inhibitory peptide” OR gip OR “anti-obesity drugs” OR “anti-obesity agents”) AND (“body composition” OR anthropometry OR “body weight” OR “electrical impedance” OR “electric impedance” OR muscle OR “magnetic resonance imaging” OR DEXA OR DXA OR “photon absorptiometry”)
