# Supplementary material for: Effect of GLP-1 receptor agonists at doses for obesity management on muscle health: systematic review and meta-analysis of randomized controlled trials (RCTs)
Source: Int J Obes (Lond). 2026 Jun 19;50(8):1638–46. doi: 10.1038/s41366-026-02118-y (PMC13461373; doi:10.1038/s41366-026-02118-y)
Supplement: Supplementary file 1 [file 41366_2026_2118_MOESM1_ESM.docx]

**Suplementary Table 1. GRADE summary of findings (SoF)**

| **Certainty assessment** | | | | | | | **№ of patients** | | **Effect** | | **Certainty** | **Importance** |
| --- | --- | --- | --- | --- | --- | --- | --- | --- | --- | --- | --- | --- |
| **№ of studies** | **Study design** | **Risk of bias** | **Inconsistency** | **Indirectness** | **Imprecision** | **Other considerations** |  |  | **Relative (95% CI)** | **Absolute (95% CI)** |  |  |
| **Change in lean body mass relative to total weight** | | | | | | | | | | | | |
| 6 | randomized trials | not serious | not serious | not serious | not serious | none | 344 | 256 | - | MD **1.81 % more** (1.1 more to 2.52 more) | ⨁⨁⨁⨁ High | CRITICAL |
| **Changes in lean body mass %** | | | | | | | | | | | | |
| 6 | randomized trials | not serious | not serious | not serious | not serious | none | 344 | 256 | - | MD **3.06% lower** (5.1 lower to 1.02 lower) | ⨁⨁⨁⨁ High | CRITICAL |
| **Changes in lean body mass (KG) (absolute change)** | | | | | | | | | | | | |
| 6 | randomized trials | not serious | not serious | not serious | not serious | none | 344 | 256 | - | MD **1.74 kg lower** (3.04 lower to 0.45 lower) | ⨁⨁⨁⨁ High | IMPORTANT |

**CI:** confidence interval; **MD:** mean difference
